# The Role of CTNNB1 in Endometrial Cancer

**DOI:** 10.1155/2022/1442441

**Published:** 2022-04-28

**Authors:** Živa Ledinek, Monika Sobočan, Jure Knez

**Affiliations:** ^1^Department of Pathology, University Medical Centre Maribor, Ljubljanska ulica 5, 2000 Maribor, Slovenia; ^2^Divison for Gynaecology and Perinatology, University Medical Centre Maribor, Ljubljanska ulica 5, 2000 Maribor, Slovenia; ^3^Department of Gynaecology and Obstetrics, Faculty of Medicine University of Maribor, Taborska ulica 8, 2000 Maribor, Slovenia

## Abstract

Endometrial cancer (EC) is the most common gynaecologic malignancy in the developed countries. Recent evidence suggests that histopathological subtyping together with molecular subgrouping can lead to more accurate assessment of the risk profile for the patient. Clinical studies suggest the currently used molecular classification improves the risk assessment of women with endometrial cancer but does not explain the differences in recurrence profiles clearly. This could be improved by novel markers. One of such are mutations in the *β*-catenin (*CTNNB1*) gene, a frequently mutated gene in endometrial cancer. This shows mutations mostly at phosphorylation sites of the *β*-catenin and almost exclusively in the endometrial subgroup of no specific molecular profile. *CTNNB1* mutations lead to alterations in the Wnt/*β*-catenin signalling pathway, involved in the carcinogenesis and progression of EC by inducing transcription of target genes, whose function is to regulate the cell cycle. Although tumours with mutations in *CTNNB1* tend to have low-risk characteristics, they are related to worse outcomes with significantly increased rate of disease recurrence and lower overall survival.

## 1. Introduction

Endometrial cancer (EC) is the most common gynaecologic malignancy in Europe and second most common worldwide with an incidence of 417 367 in 2020 [1, 2]. In 70-75% of cases, EC is diagnosed in the early stage of the disease. The average overall 5-year survival rate is 76%, and in low-risk, early-stage disease, the 5-year survival exceeds 90% [2, 3]. But even when EC is diagnosed and managed at an early stage, the disease recurs in up to 20% of cases. Unfortunately, the median survival for recurrent or metastatic disease barely exceeds 12 months [4]. Developing new tools to prevent and predict EC recurrence is therefore of critical importance to improve the management of the disease. The aim of this review is to assess the role of *CTNNB1* (*β*-catenin) in EC as a possible biomarker for risk stratification.

## 2. Molecular Classification of EC

EC is today the most commonly diagnosed gynaecological malignancy and is managed according to the European Society for Gynaecologic Oncology/European Society for Radiotherapy and Oncology/European Society for Pathology (ESGO/ESTRO/ESP) guidelines for EC [2]. Histopathologic typing of tumours is performed based on the World Health Organization (WHO) Classification of Tumours (5^th^ edition) [5], including the histopathologic type, grade, myometrial invasion, and lymphovascular space invasion (LVSI). EC is divided into the following histopathological subtypes: endometrioid adenocarcinoma, serous adenocarcinoma, clear-cell adenocarcinoma, and either undifferentiated or dedifferentiated adenocarcinoma. Tumours are additionally graded with a binary grading system as either low-grade (FIGO grade 1 and 2) or high-grade (FIGO grade 3) [2, 5].

The recent ESGO/ESTRO/ESP guidelines suggest that molecular characterisation of the tumours should be applied where available. Molecular characteristics of tumours contribute to risk stratification of both low-grade and high-grade tumours and better diagnosis reproducibility, especially of high-grade tumours [5, 6]. In high-grade EC, the prognosis further varies between the histopathological subtypes with serous tumours having significantly poorer prognosis than endometrioid carcinoma. Therefore, poor reproducibility of histopathological diagnosis can in some cases lead to erroneous risk stratification [5, 6].

WHO Classification of Tumours (5^th^ edition) lists four molecular subtypes of EC. Those are (1) *POLE* (DNA Polymerase Epsilon) ultramutated tumours, (2) mismatch repair-deficient (MMRd) tumours, (3) p53-mutant tumours (p53abn), and (4) tumours of no specific molecular profile (NSMP) [5]. Data of different research groups show that among different molecular subtypes, endometrial carcinoma falls into the NSMP group in 44-56% of cases, the MMRd group in 25-34% of cases, to the p53abn group in 8-26%, and into the POLEmut group in 3-6% of cases [7–9].

**Figure 1:**
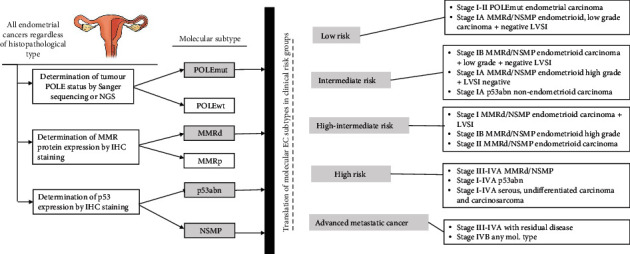
Diagram of risk stratification for patients with EC based on WHO Classification of Tumours (5^th^ edition) and ESGO/ESTRO/ESP guidelines for the management of patients with endometrial carcinoma considering molecular subtypes of EC [5, 7].

Based on described histopathological and clinical evaluation, patients are stratified into one of the prognostic risk groups as shown in Figure 1, depending also on whether the information about molecular classification of the tumour is available [2].

To compare with histopathological subtypes, endometrioid tumours are more commonly POLE-ultramutated, MMRd, or NSMP, whereas other histological subtypes of tumours fall into the category of p53-abn [10, 11]. Most endometrioid tumours have few somatic copy-number alterations (SCNAs) whereas most serous and serous-like tumours exhibit extensive SCNAs, and the extent of SCNA roughly correlates with progression-free survival [12]. However, there is substantial variability in the clinical course of the disease, associated with both morphological and molecular features of the tumour [13].

## 3. Study Selection Methods and Data Evaluation

**Figure 2:**
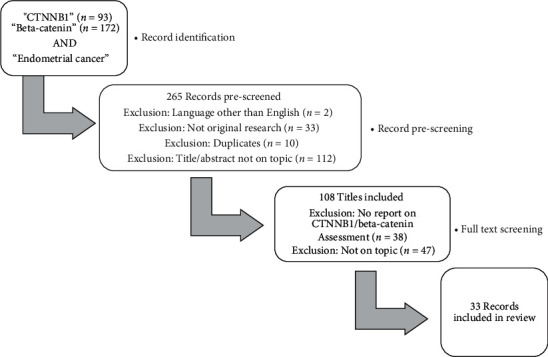
Study selection process.

A broad-spectrum literature review was performed through the Medline database. We included search terms on the *CTNNB1* gene as well as the protein beta-catenin to fully assess the available literature on endometrial cancer. The study selection process is depicted in Figure 2. Study selection was performed in November 2021.

In accordance with the aims of this review, titles were excluded if studies had not specifically focused on *CTNNB1* mutation and assessed data against this mutation (e.g., *CTNNB1* as part of a multigene molecular panel with no specific analysis pertaining *CTNNB1*), if they were focusing on therapeutics, or if they were focused on other cancer subtypes. The final selection of studies included clinical studies, diagnostic studies, as well as mechanistic and genetic studies of *CTNNB1*/beta-catenin in endometrial cancer.

In addition to the literature review, a further analysis of publicly available databases of genetic mutations in human cancer, namely, The Cancer Genome Atlas (TCGA) [14], cBioPortal for Cancer Genomics [15, 16], and Catalogue of Somatic Mutations (COSMIC), [17] was performed. Tumour samples from the TCGA database were selected if the defined primary cancer sites (in the) uterus (NOS) or corpus uteri and if they harboured any CTNNB1 mutation regardless of the tumour type or any other filters. From cBioPortal database, all studies of uterine tumours were selected and analysed for the *CTNNB1* gene. The COSMIC database for *CTNNB1* mutations was searched by filtering data to “endometrium” as the target tissue. This search resulted in 119 identified mutations from TCGA database and 349 mutations from cBioPortal. Duplicate mutations were removed in tissue samples originating from the same volunteer. Additional 135 mutations were also identified from the COSMIC database. All listed mutations were mapped based on the amino acid sequence of beta-catenin protein. List of mutations was compared to the interaction sites between *β*-catenin and other proteins. List of interactions was based on the intracellular function of beta catenin, linked to the carcinogenesis and progression of EC.

## 4. Wnt Signalling and (over) Expression of *β*-Catenin in EC

Hyperactivation of Wingless/int1 (Wnt)/*β*-catenin signalling has been implicated in tumorigenesis, tumour progression, recurrence, and chemoresistance of gynaecological malignancies [11, 18–20]. Specifically, Wnt signalling promotes metastasis and therapy resistance in ovarian cancer, plays a crucial role in tumorigenesis and recurrence in endometrial cancer, and participates in human papillomavirus (HPV) related tumorigenesis and metastasis in cervical cancer [21].

**Figure 3:**
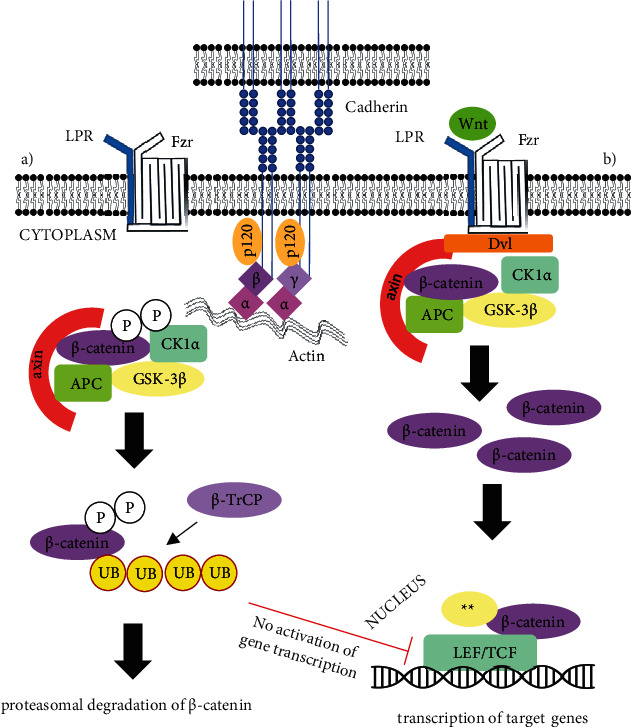
Intracellular functions of *β*-catenin. *β*-Catenin as adherent junction protein is (together with *γ*-catenin) attached to cadherin via *δ*-1 catenin or p120 protein, both are also attached to *α*-catenin, which is connected to the actin filaments of cytoskeleton. Intracellular function of Wnt/*β*-catenin pathway is shown in the presence and absence of Wnt ligand. (a) In the absence of Wnt ligand, *β*-catenin is phosphorylated by the destruction complex containing axin, adenomatous polyposis coli protein (APC), glycogen synthase kinase 3 beta (GSK-3*β*), and casein kinase 1*α* (CK1*α*), ubiquitinated by F-box/WD repeat-containing protein 1A (*β*-TrCP or Fbxw1), and targeted for proteasomal degradation. In the absence of *β*-catenin, the transcription complex T cell factor/lymphoid enhancer factor family LEF/TCF remains repressed. (b) When Wnt ligands bind to Frizzled (Fzd) receptors and lipoprotein receptor-related protein (LRP) coreceptors the latter being responsible for recruiting dishevelled (Dvl) polymers which inactivate the destruction complex leading to the accumulation of *β*-catenin in the cytosol. *β*-Catenin is translocated from the cytosol to the nucleus where it forms and active complex with LEF/TCF proteins and other histone modifying coactivators^∗∗^ leading to transcription of multiple genes, involved in the process of cell maturation and proliferation [24].

When activated, *β*-catenin-dependent Wnt signalling pathway leads to the accumulation of *β*-catenin in the cytosol and to formation of *β*-catenin complexes with T cell factor/lymphoid enhancer factor family (TCF/LEF-1) which act as transcription factors. Those complexes are afterwards translocated to the nucleus and induce transcription of responsive genes, among them are c-*MYC* (cellular protooncogene homologous to myelocytomatosis virus) and *CCND1* (cyclin D1) [21]. Proteins, encoded by those genes, function mostly as regulators of cell cycle, and their overexpression is linked to a variety of human cancers [22, 23].  Figure 3 summarizes the intracellular function of *β*-catenin in Wnt signalling pathway, also known as canonical pathway.

In a normal cell, without activation of Wnt signalling pathway, excessive cytosolic *β*-catenin is phosphorylated and later degraded by proteasomes [19, 21, 25]. However, most common mutations of *CTNNB1* gene occurring in EC are the mutations of exon 3, which encodes the N terminal region of the protein—its binding site for ubiquitin. Such mutations therefore lead to inefficient ubiquitination of *β*-catenin and subsequent failure of its degradation by proteasome. This causes accumulation of *β*-catenin in the cytoplasm of the cell and in turn activates the Wnt/*β*-catenin signalling pathway [18, 21].

## 5. CTNNB1 Mutations in EC

Among more frequent genetic mutations in EC are alterations in the catenin beta-1 or *β*-catenin (*CTNNB1*) gene, occurring in approximately 20-25% of tumours [21]. Catenins are a group of three subtypes (*α*, *β*, and *γ*) of cytoplasmic proteins, interacting with cadherins (Figure 3). Either *β*-catenin or plakoglobin (*γ*-catenin) forms a connection between the cytoplasmic region of E-, N-, and P-cadherins and *α*-catenin, which binds to cytoskeletal actin filaments (Figure 3) [26]. Therefore, *β*-catenin plays an important role in cell-to-cell adhesion, but apart from being an adherent junction protein, it also has an important role in the Wingless/int1 (Wnt) signal transduction pathway that regulates cell proliferation and differentiation [27–29].

Exon 3 mutations in *CTNNB1* are frequently missense mutations. These mutations mostly occur at phosphorylation sites of glycogen synthase kinase 3 beta (GSK-3*β*): S33, S37, and T41, phosphorylation sites of casein kinase 1*α* (CK1*α*): S45 and sites of interaction with F-box/WD repeat-containing protein 1A (Fbw1): D32 and G34 [30, 31]. They occur almost exclusively in NSMP molecular subtype of EC [12, 32]. Additionally, the presence of *CTNNB1* mutations appears to be independent of the presence of microsatellite instability, and the mutational status of *PTEN* (phosphatase and tensin homolog) and *KRAS* (Kirsten rat sarcoma viral oncogene homolog) [32–34]. Kim and Jeong summarized activating mutations of *CTNNB1* in endometrial cancer cell lines, occurring at some beforementioned and other sites: D32V, S37P, S37C, D207G, and X561_splice [31]. Although other mutations of the *CTNNB1* gene have been identified and associated with EC, Liu et al. found that Wnt/*β*-catenin signalling pathway was not enriched in cases where *CTNNB1* was mutated outside exon 3 [18].

**Figure 4:**
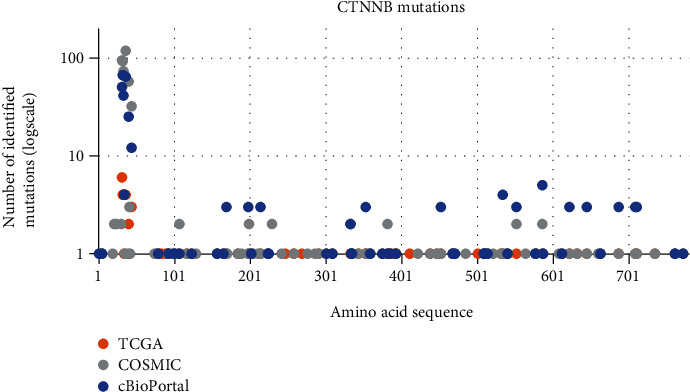
Mapped mutations of CTNNB1 in EC based on available data from the TCGA, COSIMC, and cBioPortal databases. Diagram represents the number of identified mutations (logarithmic scale) of specific amino acid in the amino acid sequence of CTNNB1 gene. Exon 3 encodes for 75 amino acids (from 5^th^ to 80^th^) where we can see a spike in the number of identified mutations in EC tumours. Other mutations are scattered throughout the whole gene, but few have been detected in more than one tumour sample.

Since there is little data about the *CTNNB1* mutations outside of exon 3, we mapped all known mutations of *CTNNB1* in EC from TCGA [14], cBioPortal [15, 16], and COSMIC [17]. As expected, and shown in Figure 4, most identified mutations of *CTNNB1* in EC tumours are located within exon 3. There are various coding and noncoding mutations outside of exon 3, among which, very few are identified as either driver (filter used by cBioPortal) or pathogenic (filter used by COSMIC) mutations as opposed to variants of unknown significance (VUS) (filter used by cBioPortal) or neutral mutations (filter used by COSMIC). To check for other possible clinically important mutations of *CTNNB1,* we cross-referenced binding sites of proteins, interacting with *β*-catenin [35], with all three beforementioned databases.

*β*-Catenin interacts with axin and APC as a part of its destruction complex [36]. It binds with axin on Thr257, Ile296, Ser250, and Trp338 residues [37], none of which were found to be mutated in EC. Its binding with APC is more complex, extending from residues 145-665 of *β*-catenin excluding the loop in armadillo repeat 10 (residues 553-560) which bind with 20 aa repeat complexes of APC [38]; thus, a point mutation of *β*-catenin would hardly affect its binding with APC. Similar to its binding with APC, interaction with *β*-catenin and other adherent junction proteins, namely, *α*-catenin and E-cadherin, forms in proximity of armadillo (ARM) repeats and includes a larger number of residues. Our genetic dataset analysis has not identified a mutation of the highly conserved Y654-*β*-catenin-D665-E-cadherin binding site [39] in any of the EC tumours.

## 6. *β*-Catenin as Adherent Junction Protein

*β*-Catenin plays an important role as an adherent junction protein (Figure 3), and its dysfunction due to mutations of *CTNNB1* could also lead to decreased cell-to-cell adhesion. This has in fact been reported in about 15% of ECs [40]. The process in which epithelial cells transform into fibroblast-like mesenchymal phenotype, thus losing their polarity and cell-to-cell contacts, is called epithelial to mesenchymal transition (EMT) [41]. In adult organisms, EMT is important for folliculogenesis and occurs as a physiological response to injury during the wound healing. In cancer cells, EMT allows the tumour cells to dissociate, migrate, and metastasize. EMT also induces cancer stem cell traits, i.e., prevents cell apoptosis and ageing, induces resistance to chemotherapy, and contributes to immunosuppression [42]. The EMT signalling pathway may be activated by several cytokines or growth factors from the local microenvironment that can be followed by the interaction with Wnt/*β*-catenin pathway. Specifically, nuclear function of *β*-catenin has been shown to promote EMT by upregulating expression of Snail Family Transcriptional Repressor 2 gene (*SNAI2*), also known as Slug [41–43]. This could mean that mutations in exon 3 of *CTNNB1*, triggering overexpression of *β*-catenin, could also predispose to the process of EMT in EC, although the connection between the two has not yet been proven. On the other hand, the process of EMT is associated to the loss of E-cadherin, due to its repressed transcription in cancer cells, causing downregulation of other adhesive epithelial markers [43]. Reduced expression of both, E-cadherin and *β*-catenin (in apical cytoplasm and not in the nucleus), has been associated with EMT [40]. However, it is not yet clear whether mutations of *CTNNB1* could in any way influence either the reduced expression of *β*-catenin or its interaction with E-cadherin.

## 7. Immunohistochemical Methods for Detection of *CTNNB1* Mutations

The tumour *CTNNB1* status is most precisely determined through the identification of specific mutations of *CTNNB1* in EC. This is done by sequencing of exon 3 [44]. Sequencing can be performed using targeted Sanger sequencing or next generation sequencing (NGS). However, NGS which was also used through the discovery process for identification of molecular subtypes of EC is costly and complex for routine clinical use [12, 45]. Since the intracellular location of *β*-catenin can be determined with immunohistochemistry (IHC), it has been suggested to use IHC methods as a proxy for *CTNNB1* mutations in EC [46].

There are currently no standardized criteria for interpretation of *β*-catenin immunostaining in EC yet. There is strong membrane *β*-catenin immunopositivity observed in normal endometrium and endometrial hyperplasia and a great variety of staining patterns in EC [47]. Florescu et al. found significant differences between intensity of membranous *β*-catenin immunopositivity and degree of tumour differentiation, invasion in the myometrium and tumour stage [40]. Such findings suggest that decreased expression of *β*-catenin, characteristic of EMT, could also be determined by IHC methods [13, 40]. Machin et al. found a correlation between exon 3 mutations of *CTNNB1* and nuclear *β*-catenin immunostaining [48]; thus, more recent studies tried to validate IHC methods as a surrogate for identification of exon 3 mutations in *CTNNB1* [44, 46, 49]. Validation studies show that specificity of *β*-catenin IHC for exon 3 mutations of *CTNNB1* is very high. In some reports, compared with detection using NGS, the specificity even reached 100% [44, 46]. Sensitivity, however, was lower (85-91%), which is possibly due to nuclear localisation of *β*-catenin, which is often focal and with different degrees of intensity [44, 46, 49]. With respect to intensity, weak nuclear expression of *β*-catenin can also be observed in proliferative phase of normal endometrium [50].

## 8. Clinical Significance of *CTNNB1* Mutations

Clinicopathological and molecular characteristics of the EC stratify ECs in prognostic groups, which guide the selection of additional therapy interventions [2, 7]. Information about the clinical value of additional prognostic risk factors such as *CTNNB1* mutations emerged later and helps to better delineate prognostic profiles, especially of FIGO stage I EC [51]. Tumours with mutations in *CTNNB1* tend to have low-grade histology, low rates of myometrial invasion with low rates of LVSI. They usually occur in women with EC at younger age, which clinically indicates a low risk of recurrence [11, 18, 20, 48]. However, mutations of *CTNNB1* gene are related to worse outcomes with significantly increased rate of disease recurrence and lower overall survival, compared to other tumours with low-grade histology, specifically in relation to mutations in exon 3 [20, 52]. This shows the necessity of integrating molecular markers to adequately assess the prognosis of tumours with low-grade histopathological characteristics to adjust therapeutical solutions and offer possibility of targeted therapy. Summary of published data on clinical outcomes of patients with *CTNNB1* mutation is presented in Table 1. These retrospective data assessments indicate that mutations in *CTNNB1* are associated with worse recurrence free survival and could possibly be prognostic for distant metastasis. Based on the frequency of *CTNNB1* mutated tumours in NSMP group (26–52%) with independent prognostic value, some authors propose *CTNNB1*–mutated ECs may be regarded as a fifth molecular subgroup [53, 54].

Dysfunction of either of its intracellular functions due to mutation of *CTNNB1* gene or other dysregulation of Wnt/*β*-catenin signalling pathway has been linked to the development and progression of EC. Costigan et al. reported recurrence rate of 30% for FIGO stage IA tumours with *CTNNB1* mutation in exon 3 in comparison to no recurrence in same stage tumours with wild-type *CTNNB1*. Also, 40% of recurrent tumours already metastasized to the lungs [49]. Stello et al. found stage 1 EC tumours with *POLE* mutation and tumours without microsatellite instability and with *CTNNB1* wild type are associated with favourable prognosis whereas tumours with microsatellite instability and *CTNNB1* exon 3 mutations are associated with intermediate prognosis based on current risk stratification system [55]. Kurnit et al. similarly found higher risk of recurrence of low grade, low stage tumours with *CTNNB1* mutations (HR: 5,97; 95 CI [2,69-13,21]; *p* < 0,001) [32]. Mutations of *CTNNB1* have also been associated with tumours in younger patients [18, 32]. *CTNNB1* status can be used to stratify FIGO stage I tumours into group with a favourable prognosis (*CTNNB1*-wild type, with a similar prognosis to POLEmut tumors) and group with an unfavourable prognosis (*CTNNB1*-mutant, with a similar prognosis to MMRd) [51].

There are two basic mechanisms related to *CTNNB1* mutations that directly influence the development and progression of the disease. First, dysregulation of cell cycle is linked to the mutations of exon 3 in *CTNNB1* gene, making it resistant to ubiquitinisation, thus leading to its intracellular accumulation and subsequently expression of various protooncogenes. Secondly, defective function of cell-to-cell adhesion influences the metastatic potential of tumour, leading to more aggressive growth of the lesion.

Understanding the role of *CTNNB1* and its transcript *β*-catenin in the carcinogenesis of the EC can lead to better risk stratification models and possible better identification of patients with potentially worse disease prognosis. Although dysfunction of *β*-catenin can influence the development and progression of EC through different molecular pathways, clinical significance of the *CTNNB1* mutations, occurring outside of exon 3, has not yet been determined. Currently, the most important issue is how to efficiently detect the *CTNNB1* mutations or its intracellular consequences in the routine diagnostic evaluation. The need for molecular characterization (specifically sequencing), which requires complex tissue preparation, makes the method costly for routine use.

Most cases of EC are still classified as nonspecific molecular profile (NSMP) [8], leaving a heterogenous group of women with different aggressiveness profiles of EC still without an exact understanding of their prognosis. Therefore, new exploration of biomarkers to aid the currently established standards is needed. There is emerging evidence on other molecular markers that play important role in determining the prognosis of more aggressive behaviour in EC. They enable additional risk stratification in already established molecular subgroups of EC [51].

Wnt signalling has been involved with some other signalling pathways, such as mammalian target of rapamycin kinase (mTOR) pathway, which regulates cell growth, proliferation, apoptosis, and angiogenesis [57, 58]. The enhancement of mTOR pathway is currently being studied as a key cause for endometrial cancer drug resistance [59]. Research of mTOR pathway has been focused on identifying potential targets for treatment of EC with biologics [59–61]. Both mTOR pathway and Wnt signalling have been linked to EMT process. Identifying key molecules of those pathways can contribute to improvement of targeted treatment options [62]. Another perspective biomarkers is *ARID1A*, a tumour suppressor gene encoding a large nuclear protein, involved in chromatin remodelling [63]. Mutation of *ARID1A* and its subsequent loss of expression can be easily assessed with immunohistochemistry [63, 64]. As prognostic marker, reduced expression of *ARID1A* has been linked to shorter progression-free survival in endometrium-related cancer as well as higher FIGO stage. It may also play an important role in transition from complex atypical hyperplasia to carcinoma [53, 64].

## 9. Conclusions

The process of carcinogenesis and progression of EC has been linked to mutations in *CTNNB1* gene, which encodes *β*-catenin, an adherent junction protein that also plays an important part in the Wnt/*β*-catenin signalling pathway. Identifying mutational status of the *CTNNB1* gene, especially in low-grade tumours, is important for more accurate risk stratification of patients and could potentially lead to better management of women. Clinical studies showed that EC with *CTNNB1* mutations has worse outcome with significantly increased rate of disease recurrence and lower overall survival. Our analysis of identified *CTNNB1* mutations shows there are currently no clinically important mutations of *CTNNB1* outside of its exon 3 that could influence the interaction of *β*-catenin with other adherent junction proteins. However, reduced expression of *β*-catenin in EC can lead to the progression of the disease and EMT. Therefore, further studies are needed to determine the role of *β*-catenin more specifically in EMT of EC and its influence on the prognosis of the disease. Exon 3 mutations lead to translocation of *β*-catenin to the nucleus which can be detected by IHC. It would still be necessary to validate clinical applicability of IHC for *β*-catenin and standardize criteria for interpretation of immunostaining.

## Figures and Tables

**Table 1:** Summary of published data on clinical impact of *CTNNB1* mutations in EC.

Study	Setting	No. of cases	Outcome
Kurnit et al. 2017 [32]	Retrospective	342	Mutations of CTNNB1 were connected to worse recurrence free survival, tumours in younger patients, low-grade histology, and lower rates of LVI, PNI, and myometrial invasion.
Imboden et al. 2020 [4]	Retrospective	41	Mutations of CTNNB1 were most common type of mutations in primary tumours with low-grade histology.
Ruz-Caracuel et al. 2021 [52]	Retrospective	218	Mutations in exon 3 of CTNNB1 were significantly associated with decreased disease-free survival in patients with low-grade, early-stage EEC.
Stelloo et al. 2016 [55]	Retrospective	834	Mutations in exon 3 of CTNNB1 were prognostic for distant recurrence of the disease.
Costigan et al. 2021 [49]	Retrospective	79	Tumours with mutations of CTNNB1 had higher rate in patients with stage IA disease at diagnosis and included distant metastases.
Moroney et al. 2019 [56]	Case-control	15	Mutations of CTNNB1 are present at significantly higher rates in recurrent stage I, grade 1 endometrial cancers.
Liu et al. 2014 [18]	Retrospective	271	Mutations in exon 3 of CTNNB1 were statistically significantly correlated with younger patients in the TCGA cohort.

## Data Availability

Pooled data on publicly available CTNNB1 mutation analysis is available by reasonable request to the corresponding author.

## References

[1] Ferlay J., Colombet M., Soerjomataram I. (2021). Cancer statistics for the year 2020: an overview. *International Journal of Cancer*.

[2] Concin N., Matias-Guiu X., Vergote I. (2021). ESGO/ESTRO/ESP guidelines for the management of patients with endometrial carcinoma. *International Journal of Gynecological Cancer*.

[3] Zadnik V., Žagar T., Lokar K., Tomšič S., Duratović Konjević A., Zakotnik B. (2021). Trends in population-based cancer survival in Slovenia. *Radiology and Oncology*.

[4] Imboden S., Tapia C., Scheiwiller N. (2020). Early-stage endometrial cancer,CTNNB1mutations, and the relation between lymphovascular space invasion and recurrence. *Acta Obstetricia et Gynecologica Scandinavica*.

[5] Herrington C. S., WHO Classification of Tumours (2020). *WHO Classification of Tumours Female Genital Tumours*.

[6] Han G., Sidhu D., Duggan M. A. (2013). Reproducibility of histological cell type in high-grade endometrial carcinoma. *Modern Pathology*.

[7] Imboden S., Nastic D., Ghaderi M. (2021). Implementation of the 2021 molecular ESGO/ESTRO/ESP risk groups in endometrial cancer. *Gynecologic Oncology*.

[8] Knez J., Sobocan M., Belak U. (2022). Pre-treatment risk assessment of women with endometrial cancer: differences in outcomes of molecular and clinical classifications in the Slovenian patient cohort. *Radiology and Oncology*.

[9] Devereaux K. A., Weiel J. J., Pors J. (2021). Prospective molecular classification of endometrial carcinomas: institutional implementation, practice, and clinical experience. *Modern Pathology*.

[10] Travaglino A., Raffone A., Gencarelli A. (2020). TCGA Classification of endometrial cancer: the place of carcinosarcoma. *Pathology Oncology Research*.

[11] Bolivar A. M., Luthra R., Mehrotra M. (2019). Targeted next-generation sequencing of endometrial cancer and matched circulating tumor DNA: identification of plasma-based, tumor-associated mutations in early stage patients. *Modern Pathology*.

[12] Levine D. A., The Cancer Genome Atlas Research Network (2013). Integrated genomic characterization of endometrial carcinoma. *Nature*.

[13] Buchynska L. G., Naleskina L., Nesina I. P. (2019). Morphological characteristics and expression of adhesion markers in cells of low differentiated endometrial carcinoma. *Experimental Oncology*.

[14] National Cancer Institute The Cancer Genome Atlas Program-National Cancer Institute. https://www.cancer.gov/about-nci/organization/ccg/research/structural-genomics/tcga.

[15] Gao J., Aksoy B. A., Dogrusoz U. (2013). Integrative analysis of complex cancer genomics and clinical profiles using the cBioPortal. *Science Signaling*.

[16] Cerami E., Gao J., Dogrusoz U. (2012). The cBio cancer genomics portal: an open platform for exploring multidimensional cancer genomics data. *Cancer Discovery*.

[17] Tate J. G., Bamford S., Jubb H. C. (2019). COSMIC: the catalogue of somatic mutations in cancer. *Nucleic Acids Research*.

[18] Liu Y., Patel L., Mills G. B. (2014). Clinical significance of CTNNB1 mutation and Wnt pathway activation in endometrioid endometrial carcinoma. *Journal of the National Cancer Institute*.

[19] Dellinger T. H., Planutis K., Tewari K. S., Holcombe R. F. (2012). Role of canonical Wnt signaling in endometrial carcinogenesis. *Expert Review of Anticancer Therapy*.

[20] Moroney M. R., Woodruff E., Qamar L. (2021). Inhibiting Wnt/beta-catenin in CTNNB1-mutated endometrial cancer. *Molecular Carcinogenesis*.

[21] McMellen A., Woodruff E. R., Corr B. R., Bitler B. G., Moroney M. R. (2020). Wnt signaling in gynecologic malignancies. *International Journal of Molecular Sciences*.

[22] Gene Gene ID: 595, CCND1, cyclin D1, Homo Sapiens (Human). https://www.ncbi.nlm.nih.gov/gene/595.

[23] Gene Gene I. D. 4609, MYC Proto-Oncogene, BHLH Transcription Factor [Homo Sapiens (Human)] - Gene - NCBI. https://www.ncbi.nlm.nih.gov/gene/4609.

[24] Zhan T., Rindtorff N., Boutros M. (2017). Wnt Signaling in Cancer. *Oncogene*.

[25] Wang Y., van der Zee M., Fodde R., Blok L. J. (2010). Wnt/*Β*-catenin and sex hormone signaling in endometrial homeostasis and cancer. *Oncotarget*.

[26] Shapiro L., Weis W. I. (2009). Structure and biochemistry of cadherins and catenins. *Cold Spring Harbor Perspectives in Biology*.

[27] Bansal N., Yendluri V., Wenham R. M. (2009). The molecular biology of endometrial cancers and the implications for pathogenesis, classification, and targeted therapies. *Cancer Control*.

[28] Schlosshauer P. W., Ellenson L. H., Soslow R. A. (2002). *β*-catenin and E-cadherin expression patterns in high-grade endometrial carcinoma are associated with histological subtype. *Modern Pathology*.

[29] Banno K., Yanokura M., Iida M., Masuda K., Aoki D. (2014). Carcinogenic mechanisms of endometrial cancer: involvement of genetics and epigenetics. *The Journal of Obstetrics and Gynaecology Research*.

[30] Gao C., Wang Y., Broaddus R., Sun L., Xue F., Zhang W. (2018). Exon 3 mutations of CTNNB1 drive tumorigenesis: a review. *Oncotarget*.

[31] Kim S., Jeong S. (2019). Mutation hotspots in the *β*-catenin gene: lessons from the human cancer genome databases. *Molecules and Cells*.

[32] Kurnit K. C., Kim G. N., Fellman B. M. (2017). CTNNB1 (beta-catenin) mutation identifies low grade, early stage endometrial cancer patients at increased risk of recurrence. *Modern Pathology*.

[33] Llobet D., Pallares J., Yeramian A. (2009). Molecular pathology of endometrial carcinoma: practical aspects from the diagnostic and therapeutic viewpoints. *Journal of Clinical Pathology*.

[34] Yeramian A., Moreno-Bueno G., Dolcet X. (2013). Endometrial carcinoma: molecular alterations involved in tumor development and progression. *Oncogene*.

[35] Gottardi C. J., Gumbiner B. M. (2001). Adhesion signaling: how *β*-catenin interacts with its partners. *Current Biology*.

[36] Kimelman D., Xu W. (2006). *β*-Catenin destruction complex: insights and questions from a structural perspective. *Oncogene*.

[37] Xing Y., Clements W. K., Kimelman D., Xu W. (2003). Crystal structure of a *β*-catenin/axin complex suggests a mechanism for the *β*-catenin destruction complex. *Genes & Development*.

[38] Xing Y., Clements W. K., Le Trong I. (2004). Crystal structure of a *β*-catenin/APC complex reveals a critical role for APC phosphorylation in APC function. *Molecular Cell*.

[39] Röper J. C., Mitrossilis D., Stirnemann G. (2018). The major *β*-catenin/E-cadherin junctional binding site is a primary molecular mechano-transductor of differentiation in vivo. *eLife*.

[40] Florescu M. M., Pirici D., Simionescu C. E. (2016). E-cadherin and *β*-catenin Immunoexpression in endometrioid endometrial carcinoma. *Romanian Journal of Morphology and Embryology*.

[41] Chiu H.-C., Li C.-J., Yiang G.-T., Tsai A., Wu M.-Y. (2019). Epithelial to mesenchymal transition and cell biology of molecular regulation in endometrial carcinogenesis. *Journal of Clinical Medicine*.

[42] Bilyk O., Coatham M., Jewer M., Postovit L.-M. (2017). Epithelial-to-mesenchymal transition in the female reproductive tract: from normal functioning to disease pathology. *Frontiers in Oncology*.

[43] Gu C. J., Xie F., Zhang B. (2018). High glucose promotes epithelial-mesenchymal transition of uterus endometrial cancer cells by increasing ER/GLUT4-mediated VEGF secretion. *Cellular Physiology and Biochemistry*.

[44] Kim G., Kurnit K. C., Djordjevic B. (2018). Nuclear *β*-catenin localization and mutation of the CTNNB1 gene: a context-dependent association. *Modern Pathology*.

[45] Kommoss S., McConechy M., Kommoss F. (2018). Final validation of the ProMisE molecular classifier for endometrial carcinoma in a large population-based case series. *Annals of Oncology*.

[46] Travaglino A., Raffone A., Saccone G. (2019). Immunohistochemical nuclear expression of *β*-catenin as a surrogate of CTNNB1 exon 3 mutation in endometrial cancer. *American Journal of Clinical Pathology*.

[47] Saegusa M., Hashimura M., Yoshida T., Okayasu I. (2001). *Β*-Catenin mutations and aberrant nuclear expression during endometrial tumorigenesis. *British Journal of Cancer*.

[48] Machin P., Catasus L., Pons C., Muñoz J., Matias-Guiu X., Prat J. (2002). CTNNB1 mutations and *β*-catenin expression in endometrial carcinomas. *Human Pathology*.

[49] Costigan D. C., Dong F., Nucci M. R., Howitt B. E. (2020). Clinicopathologic and immunohistochemical correlates of CTNNB1 mutated endometrial Endometrioid carcinoma. *International Journal of Gynecological Pathology*.

[50] Ashihara K., Saito T., Mizumoto H., Nishimura M., Tanaka R., Kudo R. (2002). Mutation of *β*-catenin Gene in endometrial cancer but not in associated hyperplasia. *Medical Electron Microscopy*.

[51] Nero C., Ciccarone F., Pietragalla A. (2021). Adjuvant treatment recommendations in early-stage endometrial cancer: what changes with the introduction of the integrated molecular-based risk assessment. *Frontiers in Oncology*.

[52] Ruz-Caracuel I., López-Janeiro Á., Heredia-Soto V. (2021). Clinicopathological features and prognostic significance of CTNNB1 mutation in low-grade, Early-Stage Endometrial Endometrioid Carcinoma. *Virchows Archiv*.

[53] De Leo A., de Biase D., Lenzi J. (2021). ARID1A and CTNNB1/*β*-catenin molecular status affects the clinicopathologic features and prognosis of endometrial carcinoma: implications for an improved surrogate molecular classification. *Cancers*.

[54] Santoro A., Angelico G., Travaglino A. (2021). New pathological and clinical insights in endometrial cancer in view of the updated ESGO/ESTRO/ESP guidelines. *Cancers*.

[55] Stelloo E., Nout R. A., Osse E. M. (2016). Improved risk assessment by integrating molecular and clinicopathological factors in early-stage endometrial cancer-combined analysis of the PORTEC cohorts. *Clinical Cancer Research*.

[56] Moroney M. R., Davies K. D., Wilberger A. C. (2019). Molecular markers in recurrent stage I, grade 1 endometrioid endometrial cancers. *Gynecologic Oncology*.

[57] Gadducci A., Cosio S., Genazzani A. R. (2011). Tissue and serum biomarkers as prognostic variables in endometrioid-type endometrial cancer. *Critical Reviews in Oncology/Hematology*.

[58] Eritja N., Yeramian A., Chen B. J. (2017). Endometrial carcinoma: specific targeted pathways. *Advances in Experimental Medicine and Biology*.

[59] Guo F., Zhang H., Jia Z., Cui M., Tian J. (2018). Chemoresistance and targeting of growth factors/cytokines signalling pathways: towards the development of effective therapeutic strategy for endometrial cancer. *American Journal of Cancer Research*.

[60] Fatima I., Barman S., Rai R., Thiel K. W., Chandra V. (2021). Targeting Wnt Signaling in Endometrial Cancer. *Cancers*.

[61] Sobočan M., Bračič S., Knez J., Takač I., Haybaeck J. (2020). The communication between the PI3K/AKT/MTOR pathway and Y-box binding protein-1 in gynecological cancer. *Cancers*.

[62] Sobočan M., Smolle M. A., Schatz C., Haybaeck J. (2020). The interplay of tumor stroma and translational factors in endometrial cancer. *Cancers*.

[63] Bosse T., Ter Haar N. T., Seeber L. M. (2013). Loss of ARID1A expression and its relationship with PI3K-Akt pathway alterations, TP53 and microsatellite instability in endometrial cancer. *Modern Pathology*.

[64] Toumpeki C., Liberis A., Tsirkas I. (2019). The role of ARID1A in endometrial cancer and the molecular pathways associated with pathogenesis and cancer progression. *In Vivo*.

